# High glucose concentrations induce TNF-α production through the down-regulation of CD33 in primary human monocytes

**DOI:** 10.1186/1471-2172-13-19

**Published:** 2012-04-14

**Authors:** Yolanda Gonzalez, M Teresa Herrera, Gloria Soldevila, Lourdes Garcia-Garcia, Guadalupe Fabián, E Martha Pérez-Armendariz, Karen Bobadilla, Silvia Guzmán-Beltrán, Eduardo Sada, Martha Torres

**Affiliations:** 1 Departamento de Investigación en Microbiología, Instituto Nacional de Enfermedades Respiratorias, Calzada de Tlalpan 4502, Sección XVI, Ciudad de México, 14080, México; 2 Clínica del Síndrome Metabólico, Instituto Nacional de Enfermedades Respiratorias, Calzada de Tlalpan 4502, Sección XVI, Ciudad de México, 14080, México; 3 Departamento de Inmunología, Instituto de Investigaciones Biomédicas, Universidad Nacional Autónoma de México, Ciudad Universitaria 04510, Ciudad de México, 70228, México; 4 Centro de Investigación Sobre Enfermedades Infecciosas, Instituto Nacional de Salud Pública, Av. Universidad 655, Cuernavaca, 62508, M&#233xico; 5 Departamento de Medicina Experimental, Universidad Nacional Autónoma de México, Ciudad Universitaria 04510, Ciudad de México, 70228, México

**Keywords:** Antioxidant, Cytokines, Monocytes, ROS, Siaglec-3, Type 2 diabetes

## Abstract

**Background:**

CD33 is a membrane receptor containing a lectin domain and a cytoplasmic immunoreceptor tyrosine-based inhibitory motif (ITIM) that is able to inhibit cytokine production. CD33 is expressed by monocytes, and reduced expression of CD33 correlates with augmented production of inflammatory cytokines, such as IL-1β, TNF-α, and IL-8. However, the role of CD33 in the inflammation associated with hyperglycemia and diabetes is unknown. Therefore, we studied CD33 expression and inflammatory cytokine secretion in freshly isolated monocytes from patients with type 2 diabetes. To evaluate the effects of hyperglycemia, monocytes from healthy donors were cultured with different glucose concentrations (15-50 mmol/l D-glucose), and CD33 expression and inflammatory cytokine production were assessed. The expression of suppressor of cytokine signaling protein-3 (SOCS-3) and the generation of reactive oxygen species (ROS) were also evaluated to address the cellular mechanisms involved in the down-regulation of CD33.

**Results:**

CD33 expression was significantly decreased in monocytes from patients with type 2 diabetes, and higher levels of TNF-α, IL-8 and IL-12p70 were detected in the plasma of patients compared to healthy donors. Under high glucose conditions, CD33 protein and mRNA expression was significantly decreased, whereas spontaneous TNF-α secretion and SOCS-3 mRNA expression were increased in monocytes from healthy donors. Furthermore, the down-regulation of CD33 and increase in TNF-α production were prevented when monocytes were treated with the antioxidant α-tocopherol and cultured under high glucose conditions.

**Conclusion:**

Our results suggest that hyperglycemia down-regulates CD33 expression and triggers the spontaneous secretion of TNF-α by peripheral monocytes. This phenomenon involves the generation of ROS and the up-regulation of SOCS-3. These observations support the importance of blood glucose control for maintaining innate immune function and suggest the participation of CD33 in the inflammatory profile associated with type 2 diabetes.

## Background

Both acute and chronic hyperglycemia are associated with inflammation [1]. Patients with newly diagnosed or established diabetes mellitus (DM) have significantly higher levels of acute-phase proteins and pro-inflammatory cytokines compared to control subjects without DM [2-5]. Monocytes isolated from patients with type 1 diabetes produce increased levels of IL-6, IL-1β and chemokines of the CXC family including IL-8 and interferon gamma-induced protein 10 (IP-10) [6]. Furthermore, monocytes from patients with DM produce higher levels of TNF-α and IL-8 in comparison to control monocytes [7-9].

TNF-α production is thought to play a role in the generation of microvascular complications associated with diabetes, e.g., by enhancing chronic eye inflammation [10,11]. In addition to triggering acute and chronic inflammation, TNF-α regulates glucose and lipid metabolism and inhibits insulin production in pancreatic beta cells [12]. TNF-α is also produced in adipose tissue.

In both clinical and experimental conditions, hyperglycemia has been shown to alter many cellular parameters. This metabolic state leads to the generation of reactive oxygen species (ROS), the activity of protein kinase C (PKC), and the expression of p38 mitogen-activated protein kinase, nuclear factor κB (NF-κB), inflammatory cytokines, and chemokines [13-15].

Diverse mechanisms have been proposed to explain how hyperglycemia contributes to inflammation. For example, PKC activity may be increased secondarily to a poorly reversible, non-enzymatic protein glycation process, which could lead to the irreversible production of advanced glycation end products (AGEs). AGEs are known to stimulate the production of inflammatory cytokines in monocytes and macrophages through the activation of a specific receptor for AGEs (RAGE) [16,17]. Additionally, hyperglycemia may stimulate the production of inflammatory cytokines by increasing the levels of peroxides and free radicals. High serum concentrations of glucose can lead to enhanced glycolysis and mitochondrial overproduction of superoxide anion (O_2_^-^) and other reactive oxygen species (ROS), which directly induce the activation of protein kinase C (PKC) and nuclear factor κB (NF-κB) [18,19]. Indeed, these transcription factors have been shown to induce the release of IL-1β and IL-6 by human monocytes cultured under high glucose conditions [20]. The secondary effects of PKC and NF- κB activation resulting from hyperglycemia can further amplify the inflammatory response, resulting in the production of the chemokine IP-10 and the up-regulated expression of TLR2 and TLR4 [6].

Although these mechanisms can partially explain the high levels of inflammatory cytokines observed under acute hyperglycemic conditions, the effects of high glucose on other regulatory molecules involved in the control of inflammatory cytokine production have not yet been identified. Low membrane expression levels of CD33 have been associated with higher levels of inflammatory cytokine production, and CD33 is expressed by myeloid progenitor cells of the bone marrow as well as peripheral blood monocytes and lymphocytes [21,22]. CD33, which is also referred to as human sialic acid-binding Ig superfamily lectin (hSiglec-3), is a member of the Siglec family that includes 11 human proteins of I-type (Ig-type) lectins with a V-set Ig-like domain and varying numbers of C2-set Ig-like domains, such as sialoadhesins (Siglec-1 and CD169), CD22 (Siglec-2), myelin-associated glycoprotein (MAG; Siglec-4), and additional members from a subgroup that contains CD33 (Siglec-3) and CD33-related Siglecs (Siglec-5 to -11) [22-25].

The regulation of cytokine production via CD33 is believed to depend on two putative conserved tyrosine-based signaling motifs contained within the cytosolic tail of CD33. These signaling motifs, known as immunoreceptor tyrosine-based inhibitory motifs (ITIMs), act as regulatory elements that inhibit signaling [22]. CD33 activity is regulated by SOCS3, which is a member of the suppressor of cytokine signaling (SOCS) protein family. The binding of SOCS3 to the phosphorylated ITIM of CD33 induces the proteosomal degradation of both molecules [26], and the reduction of CD33 surface expression on monocytes by silencing with small interfering RNA (siRNA) or antibody blockade results in the increased secretion of IL-1β, IL-8, and TNF-α [27].

Interestingly, the role of CD33 in the production of pro-inflammatory cytokines secondary to hyperglycemia has not yet been explored. Thus, the aim of the current study was to examine the effects of high glucose concentrations on the expression of CD33 and the production of cytokines in human monocytes from healthy individuals. Additionally, from patients with type 2 diabetes, the levels of CD33 expression on freshly obtained monocytes and serum cytokine levels were evaluated and compared to those from healthy individuals. Our results show that under hyperglycemic conditions, monocytes CD33 mRNA and surface protein expression was decreased, whereas TNF-α production was increased. These changes were inhibited by antioxidant pre-treatment, suggesting that the hyperglycemic-dependent decrease in CD33 expression involves the generation of oxidative stress.

## Results

The clinical characteristics of the studied subjects are summarized in Table 1. There were no significant differences in gender, age, BMI, or the levels of creatinine or LDL or HDL cholesterol between the control group and the type 2 diabetes group. The levels of glucose, HbA1c and triglycerides were significantly higher among type 2 diabetes subjects than control subjects. Most patients with type 2 diabetes had received metformin and Glibenclamide. Only one of the diabetes patients had received metoprolol, and another had been administered clonazepam and levopromazine. None of the patients had been prescribed angiotensin receptor blockers, insulin, or statins. Healthy donors did not have any infections or inflammatory diseases and did not take any medications during the study period.

**Table 1:** Demographic characteristics and clinical data of the groups

	Type 2 diabetes	Healthy	P value
n	21	26	

Gender (M/F)	8/13	6/20	0.0832

Age (years)	51.16 (10.17)	48.48 (9.42)	0.1868

BMI (kg/m^2^)	27.85 (4.64)	28.32 (2.22)	0.4579

Glucose (mg/dl)	265.30 (79.71)	96.28 (9.19)	< 0.0001 *

Creatinine (mg/dl)	0.70 (0.24)	0.76 (0.12)	0.0757

Cholesterol (mg/dl)	211.50 (37.15)	212.00 (43.44)	0.4547

Triglycerides (mg/dl)	295.50 (297.10)	198.00 (116.80)	0.0171 *

LDL cholesterol (mg/dl)	129.30 (37.29)	134.60 (34.26)	0.5

HDL cholesterol (mg/dl)	45.16 (8.96)	46.08 (12.23)	0.4019

Hb1Ac (%)	10.42 (2.04)	5.58 (0.25)	< 0.0001 *

Time since diagnosis (years)	7	-	

Data are means SD. *P values correspond to the differences between healthy and type 2 diabetes

### Diminished CD33 expression in monocytes from patients with type 2 diabetes

**Figure 1:**
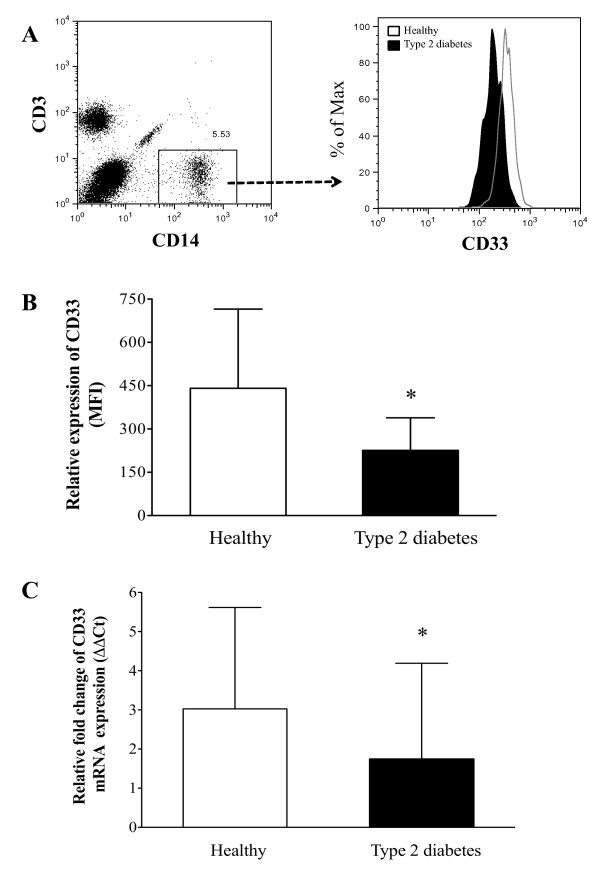
**CD33 expression in human monocytes from type 2 diabetes patients**. (A) Monocytes were stained with anti-CD3, CD14 and CD33 mAbs. At least 50,000 events were acquired for the flow cytometry analysis. CD33 expression is shown after gating for the CD3-CD14+ cells, and a histogram of CD33 expression was plotted for type 2 diabetes patients (tinted histogram) and healthy subjects (open histogram). (B) A bar graph showing the mean intensity fluorescence (MFI) data for CD33 expression in freshly isolated monocytes from patients with type 2 diabetes (n = 10) and healthy donors (n = 10). The data are presented as the mean ± SD. * P < 0.05 compared to healthy donors. (C) Monocytes from type 2 diabetes patients (n = 9) and healthy donors (n = 8) were evaluated using Taqman gene expression analysis for CD33 mRNA expression. The results were analyzed according to the ΔΔCt method, and the data are presented as the mean ± SD. * P < 0.05 compared to healthy donors.

To determine whether CD33 expression is decreased in freshly isolated peripheral monocytes from type 2 diabetes patients relative to healthy control subjects, flow cytometry and qPCR studies were performed. As shown in Figure 1, a significant decrease in the cell surface expression of CD33 was detected in monocytes obtained from patients with type 2 diabetes, as compared to those from healthy volunteers (P < 0.05) (Figure 1A and 1B).

In addition, CD33 mRNA expression was also reduced in monocytes from type 2 diabetes patients (Figure 1C). Because CD33 is a regulator of cytokine production, these findings suggest that low levels of CD33 expression could be involved in the elevated inflammatory cytokine production observed in patients with type 2 diabetes.

### Increased pro-inflammatory cytokine levels in plasma from patients with type 2 diabetes

**Figure 2:**
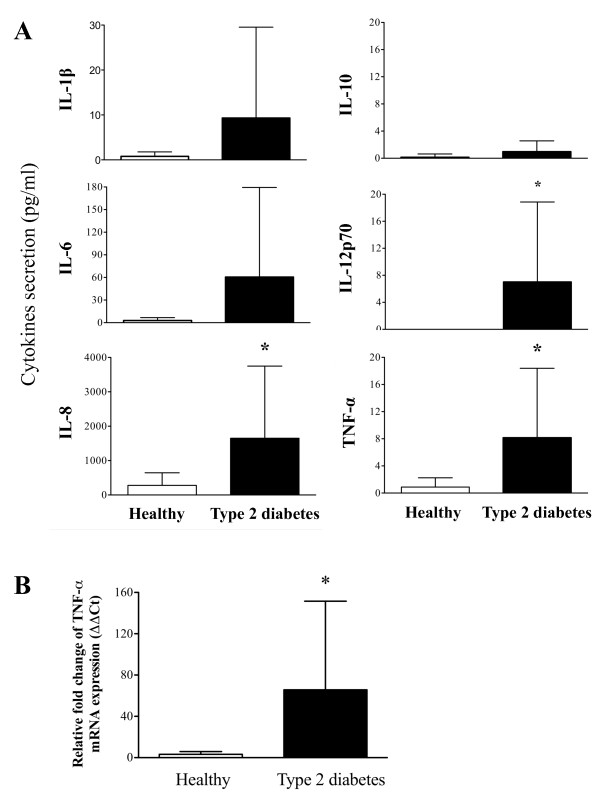
**Pro-inflammatory cytokine production in type 2 diabetes patients**. Plasma from type 2 diabetes patients and healthy controls was tested for the presence of pro-inflammatory cytokines using the CBA kit for IL-8, IL-1β, IL-6, IL-10, IL-12p70 and TNF-α. (A) The bar graphs show the quantification of these cytokines for type 2 diabetes patients (n = 14) and healthy controls (n = 10). All data are presented as the mean ± SD. * P < 0.05 compared to healthy donors. (B) The expression of TNF-α mRNA and 18 S ribosomal RNA was analyzed for monocytes collected from type 2 diabetes patients (n = 7) and healthy subjects (n = 9). The results are expressed according to the ΔΔCt method, and the data are presented as the mean ± SD. * P < 0.05 compared to healthy donors.

We next measured the levels of pro-inflammatory cytokines in the blood plasma of type 2 diabetes patients and healthy subjects. As shown in Figure 2, all of the pro-inflammatory cytokines evaluated (IL-1β, IL-6, IL-8, IL-10, IL-12p70, and TNF-α) were increased in patients with type 2 diabetes, although only the increases in TNF-α, IL-8, and IL-12 p70 were statistically significant in comparison to healthy individuals (P < 0.05) (Figure 2A). In addition, TNF-α production by monocytes was assessed by quantitative real time PCR (qPCR), and monocytes from patients with type 2 diabetes had significantly elevated levels of TNF-α mRNA than controls (P < 0.05) (Figure 2B).

### High glucose conditions down-regulate the surface expression of CD33 in cultured monocytes

**Figure 3:**
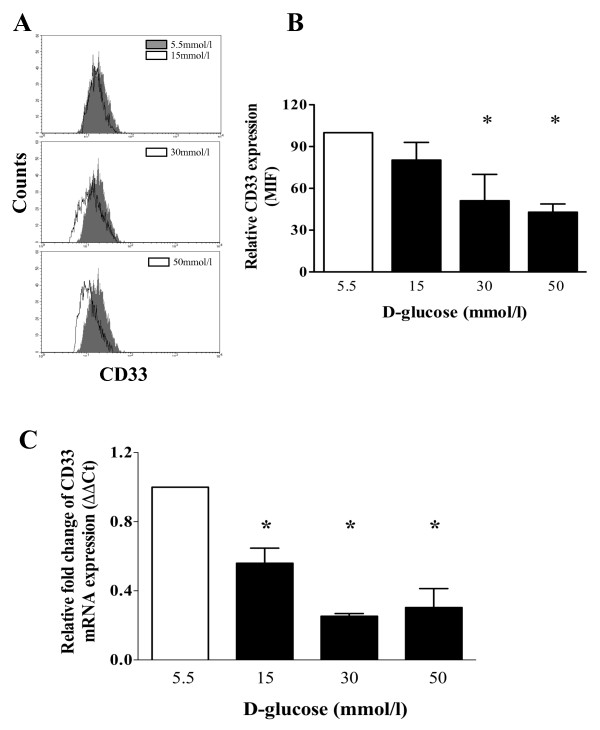
**High glucose concentrations down-regulate CD33 expression in human monocytes**. Monocytes from healthy donors were cultured in the presence of D-glucose (5.5, 15, 30, or 50 mmol/l) for 7 days. (A) Representative histograms show the expression of CD33 in monocytes cultured with 5.5 mmol/l (gray), 15, 30, or 50 mmol/l (open histogram) D-glucose. (B) Bar graphs show the MFI of CD33 in monocytes cultured under high glucose compared to those cultured in normal medium. The data are presented as the mean ± SD (n = 6). (C) Taqman gene expression mRNA analysis of CD33 and 18 S ribosomal RNA expression. The results were analyzed according to the ΔΔCt method, and all data are presented as the mean ± SD (n = 3). * P < 0.05 compared to 5.5 mmol/l D-glucose.

CD33 is known to play a role in the regulation of cytokine production, and low levels of CD33 expression have been associated with high levels of inflammatory cytokine production. Therefore, using flow cytometry, we measured the relative levels of CD33 surface expression on peripheral monocytes that were isolated from healthy volunteers and cultured in the presence of low or high concentrations of D-glucose. As shown in Figures 3A and 3B, culturing monocytes with 30 or 50 mmol/l D-glucose for 7 days induced a significant decrease in CD33 expression on the cell surface, compared to culture conditions containing 5.5 mmol/l D-glucose (P < 0.05). CD33 mRNA expression was also decreased in monocytes cultured for 7 days under high glucose conditions. As shown in Figure 3C, CD33 mRNA expression was significantly reduced in monocytes cultured in 15, 30 or 50 mmol/l D-glucose, compared to those cultured under normal glucose conditions (5.5 mmol/l D-glucose) (P < 0.05).

### High glucose conditions induce pro-inflammatory cytokine production

**Figure 4:**
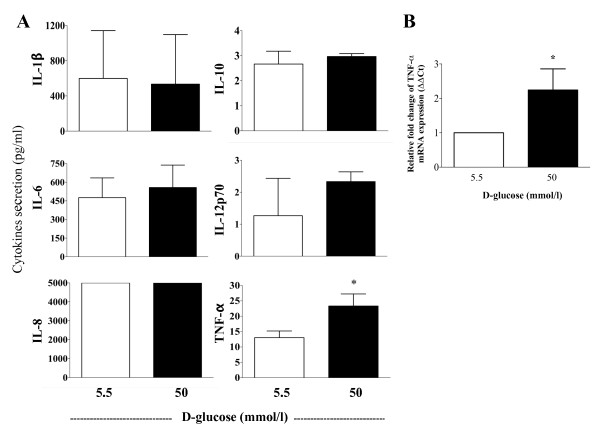
**Effect of high glucose concentrations on pro-inflammatory cytokine production**. Supernatants from monocytes cultured in the presence of 5.5 and 50 mmol/l D-glucose for 7 days were used for the quantification of IL-8, IL-1β, IL-6, IL-10, IL-12p70 and TNF-α protein levels. (A) The bar graphs represent the quantification of cytokine production (n = 3). (B) The expression of TNF-α mRNA from monocytes cultured in high glucose. TNF-α mRNA and 18 S ribosomal RNA expression was analyzed by qPCR, and the results are expressed according to the ΔΔCt method. The data are presented as the mean ± SD for all graphics (n = 3). * P < 0.05 compared to treatment with 5.5 mmol/l D-glucose.

Previous studies have shown that high glucose concentrations *in vitro *induce the production of greater amounts of IL-6 [20], which is a cytokine that can regulate CD33 expression [28]. Therefore, we evaluated the long-term effects (7 days) of high glucose conditions on the levels of interleukin-1β (IL-1β), interleukin-6 (IL-6), interleukin-8 (IL-8), interleukin-10 (IL10), interleukin-12p70 (IL-12p70), and tumor necrosis factor (TNF-α) released into the supernatants of cultured monocytes using flow cytometry. In addition, we examined the levels of cytokine mRNA using qPCR. Concentrations of 15 and 30 mmol/l D-glucose did not induce the production of pro-inflammatory cytokines (data not shown). However, 50 mmol/l D-glucose significantly induced both TNF-α secretion (P < 0.05) and TNF-α mRNA expression, compared to 5.5 mmol/l D-glucose (P < 0.05) (Figure 4A and 4B). In addition, an increase in IL-12 p70 expression was observed, although it did not reach statistical significance. The levels of IL-1β, IL-6 and IL-10 were not increased under these conditions. These results suggest that the low levels of CD33 expression observed in monocytes cultured in high concentrations of D-glucose were not the result of autocrine IL-1β, IL-6, IL-8 or IL-10 production. Nonetheless, the reduction in CD33 mRNA and cell surface protein expression may be associated with high levels of TNF-α production.

### TNF-α production is increased in monocytes cultured under high glucose conditions

**Figure 5:**
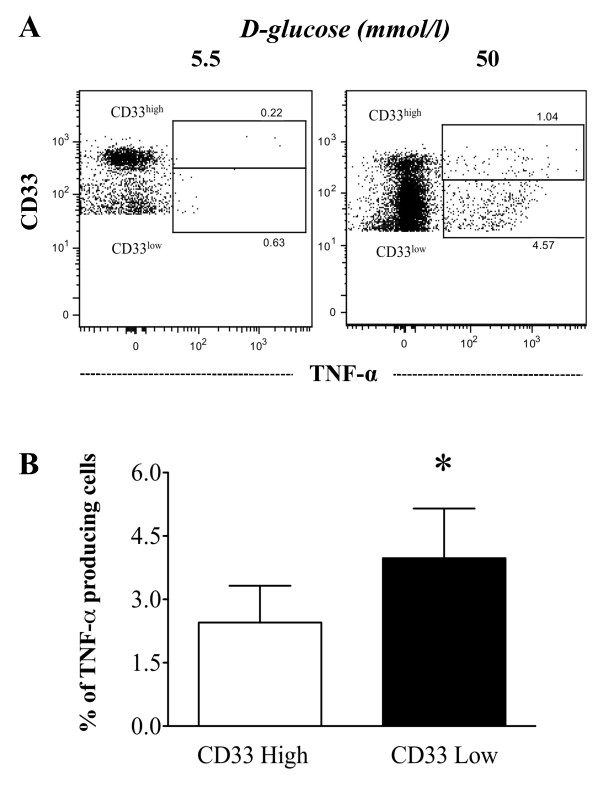
**TNF-α production in CD33^low^and CD33^high^monocytes**. Monocytes from healthy donors were cultured in the presence of glucose for 7 days and were then stained with anti-human CD33 and anti-human TNF-α antibodies. (A) Representative dot-plots show the percentages of TNF-α-producing CD33^low^ and CD33^high^ monocytes that were cultured with either 5.5 mmol/l (left) or 50 mmol/l (right) D-glucose (B) The bar graph summarizes the levels of TNF-α production by CD33^low^ (white) and CD33^high^ (black) monocytes cultured with 50 mmol/l D-glucose (n = 4). The data are expressed as the mean ± SD. * P < 0.05 as compared to the TNF-α production by CD33^low^ and CD33^high^

To investigate whether the observed increase in TNF-α production was mediated by an effect of high glucose concentrations on CD33 expression in monocytes, we assessed the production of TNF-α in monocytes expressing low or high levels of CD33. As shown in Figure 5A, 50 mmol/l D-glucose increased the relative percentage of CD33^low ^monocytes, thereby decreasing the fraction of CD33^high ^cells. However, both populations demonstrated increased TNF-α production. Thus, these results indicate that *in vitro *hyperglycemia induces the overproduction of TNF-α in both CD33^low ^and CD33^high ^monocytes. However, the frequency of TNF-α-producing cells was significantly higher in the CD33^low ^subset (Figure 5B).

### CD33 down-regulation and TNF-α production is prevented by α-tocopherol treatment

**Figure 6:**
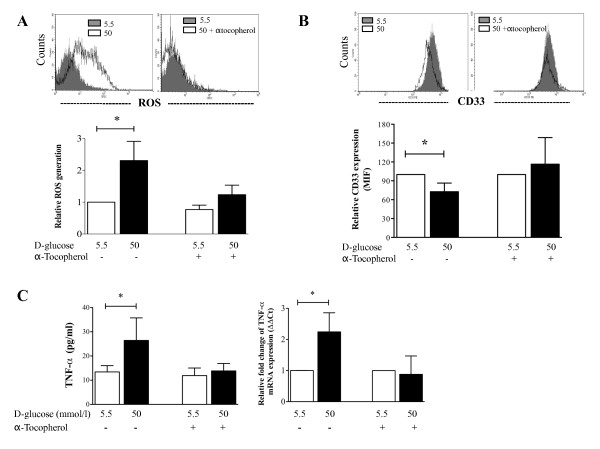
**The effect of α-tocopherol treatment on monocyte CD33 expression andTNF-α production**. Monocytes from healthy donors were cultured in 50 mmol/l D-glucose either with or without α-tocopherol for 7 days. (A) Representative histogram of ROS generation from monocytes cultured with 5.5 mmol/l (gray) or 50 mmol/l D-glucose (open histogram) without α-tocopherol (left) or with α-tocopherol (right). The bar graph shows relative ROS generation (n = 6). (B) A representative histogram showing CD33 expression from monocytes cultured without (left) or with α-tocopherol (right). The bar graph shows the relative CD33 expression levels (n = 3). (C) Left panel, the production of TNF-α, as measured by flow cytometry. Right panel, relative fold change of TNF-α mRNA expression with and without α-tocopherol treatment (n = 3). All data are expressed as the mean ± SD. * P < 0.05 compared to treatment with 5.5 mmol/l D-glucose.

The generation of ROS resulting from high concentrations of glucose is believed to contribute to hyperglycemia-induced inflammatory responses. Thus, we assessed whether ROS generation was involved in the production of TNF-α and CD33 expression by human monocytes cultured under high glucose conditions. As shown in Figure 6A, the generation of ROS by glucose was dose-dependent, and this effect was prevented by co-incubation with α-tocopherol, a compound known to reduce ROS generation and TNF-α production [29,30]. Additionally, α -tocopherol treatment prevented the decrease in surface CD33 expression by monocytes (Figure 6B), the up-regulation of TNF-α mRNA expression, and the secretion of TNF-α induced by high glucose conditions (Figure 6C). Thus, these results suggest that ROS generation induced by high glucose concentrations is responsible for the down-regulation of CD33 expression and spontaneous production of TNF-α.

### High glucose concentrations induce the expression of suppressor of cytokine signaling 3 (SOCS3) in monocytes

**Figure 7:**
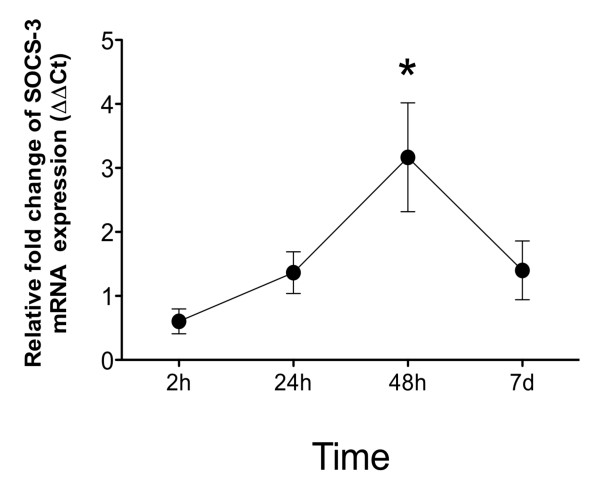
**Expression of SOCS3 mRNA in monocytes**. Monocytes from healthy donors were cultured in 5.5 or 50 mmol/l D-glucose for 2, 24, or 48 hours or 7 days, and the expression levels of SOCS3 mRNA and 18 S ribosomal RNA were evaluated using the Taqman gene assay. Gene expression was normalized to that of the housekeeping gene, and the results are expressed according the ΔΔCt method as the relative fold-change with respect to cells treated with 5.5 mmol/l D-glucose. The data are presented as the mean ± SD (n = 3). * P < 0.05.

Recent studies have demonstrated that high glucose concentrations increase the expression of SOCS3 mRNA in mononuclear cells [31,32]. Furthermore, SOCS3 has been shown to induce proteosomal degradation of CD33 in adherent monolayers of human cells [26]. Thus, we evaluated SOCS3 mRNA expression in monocytes cultured under hyperglycemic conditions for 2, 24, 48 h and 7 days. As shown in Figure 7, the levels of SOCS3 mRNA increased after 48 h of culture in the presence of high glucose concentrations (50 mmol/l). Although the levels of SOCS3 mRNA had decreased after 7 days of culture, the levels detected at this time remained greater than those in monocytes cultured in 5.5 mmol/l D-glucose. These results suggest that the down-regulation of cell surface CD33 expression mediated by high glucose concentrations may be regulated by increased levels of SOCS3 protein.

## Discussion

The primary finding of our study is that CD33 cell surface protein and mRNA expression levels are significantly reduced in monocytes from patients with type 2 diabetes. The analysis of the plasma cytokine profile of patients with type 2 diabetes showed that pro-inflammatory cytokine levels were increased, although only the levels of IL-8, IL-12, and TNF-α were significantly increased when compared to healthy volunteers. These results suggest that the elevated levels of pro-inflammatory cytokines in the serum of patients with type 2 diabetes could be related to the down-regulated expression of CD33. Increased levels of pro-inflammatory cytokines in the plasma of patients with type 2 diabetes have been widely reported [5,9,33]. On the other hand, spontaneous production of TNF-α, IL-6, and IL-8 was observed in human monocytes treated with anti-CD33 or by decreasing CD33 surface expression by RNA interference [27]. Although diverse mechanisms have been proposed to explain the increase of inflammatory cytokines in patients with type 2 diabetes, to our knowledge, this is the first study to describe an association between CD33 and the inflammatory cytokine profile in type 2 diabetes patients.

This study also demonstrated that high glucose concentrations *in vitro *resulted in decreased expression of CD33 protein and mRNA in human monocytes from healthy donors.

In addition, we observed a significant increase in levels of TNF-α present in the supernatants of monocytes cultured under high glucose conditions (50 mmol/l D-glucose), although this increase was not observed for other cytokines. Increased levels of TNF-α mRNA from monocytes cultured with 33 mmol/l glucose have previously been described for healthy individuals [3]. In addition, we did not detect an effect of high glucose concentrations on the production of IL-1β or IL-6 in monocytes. However, previous studies have reported inconsistent results; some have shown that hyperglycemia increases the production of IL-1β and IL-6 in the THP-1 cell line, whereas others have shown that hyperglycemia only induces the production of IL-6 in primary human monocytes [20,34]. In addition, other studies have demonstrated reduced IL-1β levels in RAW264 murine macrophages exposed to 8-20 mmol/l D-glucose [35]. These differences may be related to differences in the cell types, glucose concentrations or lengths of culture time used to measure cytokine levels. Other authors reported that high glucose concentration and LPS treatment act synergistically for stimulate the secretion of inflammatory cytokines in peripheral mononuclear cells from humans [36,37]. Consistent with the formerly mentioned data from other authors, our results presented here serve to extend previous knowledge of the role of high glucose concentrations on the promotion of inflammation by demonstrating the *in vitro *effects of high glucose concentrations on TNF-α production by human monocytes.

We also presented evidence demonstrating that high glucose concentrations *in vitro *could increase the proportion of CD33^low ^monocytes and reduce the proportion of CD33^high ^monocytes, although TNF-α production was increased in both cell populations. These results support the idea that hyperglycemia leads to an increase in TNF-α production through a CD33-mediated mechanism, although there are likely additional mechanisms involved in production of TNF-α that are beyond the scope of this study. Furthermore, the spontaneous production of IL-6 by CD33^-/low^plasmacytoid dendritic cells from patients with diabetes without atherosclerotic complications has been reported [38]. These findings suggest that the increased production of pro-inflammatory cytokines in patients with type 2 diabetes may be partially associated with the subpopulation of CD33^low ^monocytes.

The precise mechanisms by which hyperglycemia down-regulates CD33 expression have not yet been elucidated, although the generation of ROS by high glucose concentrations is believed to contribute to hyperglycemia-induced inflammatory responses [19,29,30]. Thus, we explored the association between ROS generation and CD33 expression in monocytes cultured under high glucose concentrations and treated with α-tocopherol. The results showed that α-tocopherol decreased ROS generation and prevented the effect of high glucose on CD33 expression. This result supports the idea that the oxidative stress generated by high glucose concentrations contributes to the down-regulation of CD33.

We observed an inhibition of TNF-α production in monocytes that were cultured under conditions of high glucose and were treated with α-tocopherol. This result indicates that ROS generation is involved in the TNF-α production by human monocytes cultured under high glucose conditions. Thus, the low expression of CD33 and the inhibition of TNF-α production in monocytes cultured under high glucose concentrations are primarily related to ROS generation. Therefore, we propose that ROS generation induced by high glucose conditions directly induces the down-regulation of CD33 expression. Alternatively, ROS generation could induce the production of pro-inflammatory cytokines that could then regulate the expression of CD33. A study by Shamsasenjan et al. postulated that IL-6 down-regulates CD33 expression in myeloma cells [28]. However, in the current study, IL-6 production was not increased in the supernatants of monocytes cultured under high glucose conditions, and therefore, IL-6 is likely not the mechanism responsible for CD33 regulation under hyperglycemic conditions.

In this study, we also showed that high glucose concentrations could up-regulate the expression of SOCS3 mRNA in human monocytes, suggesting that this molecule may regulate the levels of monocyte CD33 expression. This hypothesis is consistent with results showing that SOCS3 could contribute to CD33 degradation in peripheral monocytes [26]. Recently, it was reported that glucose ingestion induces the over-expression of SOCS3 in peripheral monocytes [31,32,39]. Interestingly, SOCS3 expression is induced by TNF-α and could therefore represent a feedback mechanism for inflammation associated with CD33 regulation [40,41]. However, further studies are needed to assess whether TNF-α production regulates SOCS3 expression and its effect on CD33 expression.

Our study had limitations, the most critical of which was the limited ability of our *in vitro *model to recapitulate what occurs in patients with type 2 diabetes. Nonetheless, we demonstrated that a significant increase in TNF-α production and decrease in CD33 protein and mRNA expression were induced by high concentrations of glucose (30-50 mmol/l). A concentration of 50 mmol/l is equivalent to 900 mg/dl of blood glucose, which is a concentration that is rarely attained in type 2 diabetes patients is much greater than the mean value found in the diabetes patients included in this study (265.3 ± 79.71 mg/dl or 14.73 ± 4.42 mmol/l blood glucose). Hence, it is possible that the glucose sensitivity of TNF-α production associated with CD33 expression is greater *in vivo *than *in vitro*. However, this increased level of sensitivity may occur if other factors *in vivo *could potentiate glucose-induced ROS generation. Further studies are required to examine this possibility. The increase in TNF-α associated with the down-regulation of CD33 expression presented here constitutes an interesting *in vitro *model to further investigate the molecular processes involved in the modulation of inflammation by glucose.

## Conclusion

We conclude that hyperglycemic conditions induce the down-regulation of CD33, which triggers the secretion of the pro-inflammatory cytokine TNF-α by monocytes. The mechanisms underlying the regulation of the TNF-α release induced by CD33 down-regulation may involve the generation of oxidative stress and the over-expression of SOCS3.

## Methods

### Participants

Twenty-one patients with type 2 diabetes and twenty-six healthy subjects were enrolled in this study. Type 2 diabetes patients were invited to participate in the study at the metabolic syndrome clinic of the National Institute of Respiratory Diseases (INER) in Mexico City. Patients with type 2 diabetes were diagnosed according to the criteria of the American Diabetes Association (diagnosis and classification of diabetes mellitus) [42]. Sixty milliliters of heparinized human peripheral venous blood was obtained from consenting individuals. The study was approved by the Institutional Review Board of the National Institute of Respiratory Diseases.

### Monocyte isolation

Mononuclear cells (PBMCs) were obtained from whole blood by centrifugation using Lymphoprep^® ^(NycomedPharma, Oslo, Norway) [43]. Monocytes were enriched by adherence and positive selection using MACS^® ^magnetic beads coupled to anti-human CD14 antibodies (Miltenyi Biotech, Auburn, CA), according to the manufacturer's recommendations. Purity was assessed by conventional flow cytometry using anti-human CD14 antibodies; the cell preparations were routinely > 95% monocytes. Monocyte viability was evaluated using the (4,5-dimethylthiazol-2-yl)-2,5-diphenyltetrazolium bromide (MTT) reduction assay [44], and cell viability was typically above 95%.

### Media and cell culture

Monocytes were cultured in RPMI 1640 (Cambrex, Walkersville, MD) supplemented with 50 μg/ml gentamicin sulfate, 2.0 mmol/l L-glutamine and 10% heat-inactivated pooled human serum (Gemini Bioproducts, Sacramento, CA) at 37°C in a 5% CO2 atmosphere. Control medium contained 5.5 mmol/l D-glucose, and high glucose conditions were achieved by supplementation with D-glucose (Sigma, St. Louis, MO) to obtain final concentrations of 15, 20, 30, or 50 mmol/l D-glucose. CD33 expression at the cell surface was examined using flow cytometry. Monocytes from healthy volunteers were cultured with or without high concentrations of glucose (15, 20, 30, or 50 mmol/l) for 7 days at 37°C and 5% CO2 in ultra-low adherence 24-well cell culture plates. Cells were then mechanically detached by pipetting, and cell viability was typically greater than 95%, as assessed by the MTT assay. The cells were then stained using phycoerythrin (PE)-labeled anti-human CD33 and PerCP-labeled anti-human CD3 antibodies (BD Biosciences, San Jose, CA, USA) or isotype controls. Fresh monocytes from type 2 diabetes patients were also stained. Cells were fixed with 1% paraformaldehyde, and CD33 surface expression was analyzed by flow cytometry. The results are expressed as the mean fluorescence intensity (MFI) of 20,000 acquired events. CellQuest software version 3.1 (BD Biosciences) was used for the analysis of the samples.

### Total RNA extraction, cDNA synthesis and gene expression analysis by qPCR

Monocytes from healthy volunteers were cultured with 5.5, 15, 30, or 50 mmol/l D-glucose for 7 days, and the supernatants were collected and stored at -20°C for the cytokine measurements. Cell viability was routinely above 95%, as assessed by the MTT assay. Monocytes (5 × 10^5 ^cells) were lysed in RTL buffer (Qiagen, Germantown, MD), and total RNA was column-isolated according to the manufacturer's recommendations (RNase mini kit, Qiagen). RNA was also purified from freshly isolated monocytes of patients with type 2 diabetes. cDNA was prepared using a SuperScript First-Strand Synthesis System for RT-PCR kit (Invitrogen, Carlsbad, CA). Briefly, RNA was mixed with 10 mMdNTPs and random hexamers and incubated at 65°C for 5 minutes. The samples were then mixed with a Reverse Transcriptase Mix (10× Reverse Transcriptase buffer, 25 mM MgCl_2_, RNaseOUT recombinant ribonuclease inhibitor and 0.1 M DTT). cDNA was synthesized using the SuperScript II RT enzyme, and samples were placed in an iCycler (Bio-Rad, Hercules, CA) with the following cycling conditions: 25°C for 10 min, 42°C for 50 min and 70°C for 10 min. The samples were also treated with RNaseH.

The levels of CD33, IL-1β, IL-6 and TNF-α mRNA were measured and normalized to that of the 18 S ribosomal RNA housekeeping gene. Real-time PCR reactions were performed in duplicate wells, according to the protocols for Taqman gene assays for CD33 (Hs00233544_m1), TNF-α (Hs01000485_m1), IL-1β (IHs0174097_m1), IL-6 (Hs00985639_m1), SOCS-3 (Hs01000485_g1), and 18 S ribosomal RNA (Applied Biosystems, Carlsbad, CA). Briefly, a universal master mix was added to the target gene primers and probes (CD33, SOCS3, IL-1β, IL-6, TNF-α, and 18 S ribosomal RNA), DEPC water and cDNA samples (diluted 1:5), and 25 μl from each reaction was added to a single well of a 96-well optical reaction plate, which was then sealed with optical adhesive film. Amplification reactions were performed using an ABI Prism 7700 Sequence Detection System (Applied Biosystems) with the following conditions: 2 minutes at 50°C, 10 minutes at 95°C, and 40 cycles consisting of 30 seconds at 95°C and one minute at 62°C. Gene expression was normalized to that of 18 S ribosomal RNA, and the results were analyzed according to the ΔΔCt method and reported as the fold-change in comparison to samples treated with 5.5 mmol/l D-glucose.

### Pro-inflammatory cytokine production

Monocytes from healthy volunteers were cultured under low or high glucose conditions (5.5 and 50 mmol/l D-glucose, respectively) for 7 days at 5 × 10^5 ^cells/well in 24-well cell culture plates. Supernatants were collected and frozen at -20°C for cytokine quantification. Secreted cytokines were measured in the supernatants using a pro-inflammatory cytometric bead array (CBA) kit (BD Biosciences), according to the manufacturer's recommendations. Cytokine concentrations were also quantified in the blood plasma of patients with type 2 diabetes. The cytokines measured included IL-1β, IL-6, IL-8, IL-10, IL-12p70 TNF-α. Cell Quest software was used for the sample acquisition, and the data were analyzed using the Cytometric Bead Array software (CBA, BD Biosciences). Values were extrapolated from a standard concentration curve and are expressed as pg/ml.

### Monocyte culture in the presence of high glucose concentrations and α-tocopherol

α-tocopherol was used as an antioxidant treatment in monocyte cultures from healthy volunteers. α-tocopherol was diluted in pooled human serum (PHS) at a concentration of 100 μmol/l and was protected from light. Monocytes (5 × 10^5 ^cells) were pre-incubated for 20 minutes with 100 μmol/l α-tocopherol. Culture medium with the high glucose concentration (50 mmol/l D-glucose) was then added to simulate hyperglycemic conditions *in vitro*. Cells were cultured for 7 days and were then assessed for the production of reactive oxygen species (ROS) and the expression of CD33.

### Determination of reactive oxygen species (ROS) generation

The fluorescent marker 5-(and 6-) carboxy-2,7-dichlorodihydrofluorescein diacetate (carboxy-HZDCF-DA; Molecular Probes Inc., Eugene, OR, USA) was used to assess ROS production. Carboxy-HZDCF-DA enters the cell and is deacetylated, oxidized by reactive oxygen and nitrogen species and converted to the fluorescent compound 5-(and-6-) carboxy-2,7-dichlorofluorescein (carboxy-DCF). Following incubation under high glucose or high glucose plus α -tocopherol conditions, the cells were loaded with 10 μMcarboxy-HZDCF-DA for 30 min at 37°C in an atmosphere of 5% CO_2_. The cells were then stained using PE-labeled anti-human CD33 and PerCP-labeled anti-human CD3 antibodies or isotype controls. Carboxy-DCF fluorescence was evaluated in CD33-positive cells by flow cytometry. The results for ROS production are expressed as the carboxy-DCF mean fluorescence intensity (MFI) of 20,000 acquired events.

### Statistical analysis

The data are expressed as the means ± SD. Nonparametric data were analyzed using the Mann Whitney test. For multiple conditions and repeated measures, the Friedman test and Dunn's test were performed. The data were analyzed using Prism software (GraphPad Prism Software version 5.0), and statistical significance was set at P < 0.05.

## Authors' contributions

YG performed the experiments, data analysis and interpretation and wrote the manuscript. MTH, KB and SGB collected the data. GS, LGG and EMPA made valuable contributions to the study design, analysis and interpretation of data and the written manuscript. GF performed the clinical analysis/discussion, and ES participated in the study design and the interpretation of the data. MT participated in the study design and wrote the manuscript. All authors reviewed and approved the final manuscript.
